# An optimized Factor H-Fc fusion protein against multidrug-resistant *Neisseria gonorrhoeae*

**DOI:** 10.3389/fimmu.2022.975676

**Published:** 2022-08-30

**Authors:** Jutamas Shaughnessy, Aleyo Chabeda, Y. Tran, Bo Zheng, Nancy Nowak, Carolynn Steffens, Rosane B. DeOliveira, Sunita Gulati, Lisa A. Lewis, James Maclean, John A. Moss, Keith L. Wycoff, Sanjay Ram

**Affiliations:** ^1^ Division of Infectious Diseases and Immunology, University of Massachusetts Chan Medical School, Worcester, MA, United States; ^2^ Planet Biotechnology, Inc., Hayward, CA, United States; ^3^ Oak Crest Institute of Science, Monrovia, CA, United States

**Keywords:** *Neisseria gonorrhoeae*, gonorrhea, factor H, immunotherapeutic, Fc fusion protein, *Nicotiana benthamiana*, complement

## Abstract

Novel therapeutics against the global threat of multidrug-resistant *Neisseria gonorrhoeae* are urgently needed. Gonococci evade killing by complement by binding factor H (FH), a key inhibitor of the alternative pathway. FH comprises 20 short consensus repeat (SCR) domains organized as a single chain. Gonococci bind FH through domains 6 and 7, and C-terminal domains 18 through 20. Previously, we showed that a chimeric protein comprising (from the N- to C-terminus) FH domains 18-20 (containing a point mutation in domain 19 to prevent lysis of host cells) fused to human IgG1 Fc (called FH*/Fc1) killed gonococci in a complement-dependent manner and reduced the duration and bacterial burden in the mouse vaginal colonization model of gonorrhea. Considering the *N. gonorrhoeae*-binding FH domains 18-20 are C-terminal in native FH, we reasoned that positioning Fc N-terminal to FH* (Fc1/FH*) would improve binding and bactericidal activity. Although both molecules bound gonococci similarly, Fc1/FH* displayed a 5-fold lower IC50 (the concentration required for 50% killing in complement-dependent bactericidal assays) than FH*/Fc1. To further increase complement activation, we replaced human IgG1 Fc in Fc1/FH* with Fc from human IgG3, the most potent complement-activating IgG subclass, to obtain Fc3/FH*. Bactericidal activity was further increased ~2.3-fold in Fc3/FH* compared to Fc1/FH*. Fc3/FH* killed (defined by <50% survival) 45/45 (100%) diverse PorB1B-expessing gonococci, but only 2/15 PorB1A-expressing isolates, in a complement-dependent manner. Decreased Fc3/FH* binding accounted for the limited activity against PorB1A strains. Fc3/FH* was efficacious against all four tested PorB1B gonococcal strains in the mouse vaginal colonization model when administered at a dose of 5 µg intravaginally, daily. Furthermore, Fc3/FH* retained bactericidal activity when reconstituted following lyophilization or spray-drying, suggesting feasibility for formulation into intravaginal rings. In conclusion, Fc3/FH* represents a promising prophylactic immunotherapeutic against multidrug-resistant gonococci.

## Introduction

Gonorrhea is caused by the Gram-negative bacterium *Neisseria gonorrhoeae*. Each year about 87 million new cases of gonorrhea occur worldwide (1). Common clinical manifestations of gonorrhea include cervicitis, urethritis, proctitis, and conjunctivitis. Serious sequelae in woman include infertility, ectopic pregnancy and chronic pelvic pain. Concomitant infection with HIV and gonorrhea enhances the rate of HIV transmission (2–4). Over the years *N. gonorrhoeae* has become resistant to almost every antibiotic that has been used for treatment (5, 6). The recent emergence of azithromycin-resistant isolates in several countries (7–10) has led the CDC to no longer include azithromycin as a treatment for gonorrhea; ceftriaxone as a single agent and at a higher dose (500 mg, single dose for uncomplicated infections) is now the only recommended first-line treatment (11).

In light of rapidly emerging multidrug-resistant *N. gonorrhoeae* worldwide, development of safe and effective vaccines and novel therapeutics against gonorrhea is a high priority (12). Targeting bacterial virulence mechanisms is an attractive approach for developing new and effective therapeutics because resistance, if it were to develop, would lead to the microbe incurring a fitness cost. Inhibiting complement is important for gonococcal virulence (13–15). One of several complement evasion mechanisms possessed by gonococci is binding factor H (FH), a key inhibitor of the alternative pathway of complement (16). FH comprises 20 short consensus repeat (SCR) domains organized in a head-to-tail manner as a single chain (17). *N. gonorrhoeae* binds FH through domains 6 and 7 (18, 19) and the C-terminal domains 18 through 20 (16, 20). We previously designed an anti-infective immunotherapeutic molecule by combining the *N. gonorrhoeae*-binding C-terminal domains 18-20 of FH, with a D to G mutation at position 1119 in FH (termed FH*) to minimize complement activation on host cells while retaining binding to *Ng*, with human IgG1 Fc (the antibody-like effector region of the modified molecule [termed FH*/Fc1]) (21). We showed that FH*/Fc1 possessed complement-dependent bactericidal activity against gonococci *in vitro* and shortened the duration and diminished bacterial loads in the mouse model of vaginal colonization (21). The function of FH*/Fc1 was further optimized by adding linkers between the FH* and Fc domains (22). ‘Linker-optimized’ FH*/Fc1 molecules, expressed in *Nicotiana benthamiana* (tobacco plants) were active *in vitro* and when administered intravaginally, accelerated clearance of bacteria in the mouse vaginal colonization model of gonorrhea (22). Here, we optimize the function of FH*/Fc fusion proteins by changing the location of the FH* relative to Fc and Fc subclass switching.

## Materials and methods

### Bacterial strains

Strains of *N. gonorrhoeae* used in this study and their relevant characteristics are listed in **Supplemental Table S1**.

### Expression and purification of FH/Fc fusion proteins in tobacco plants

A nucleotide sequence encoding human FH SCR18-20 (GenBank accession no. NP_000177) (aa 1048-1231, incorporating the D1119G mutation (23)), designed to employ optimal codon usage for expression in *Nicotiana benthamiana*, was synthesized by GENEWIZ (South Plainfield, NJ). This sequence (and the encoded protein fragment) was designated FH*.

The synthetic FH* sequence was cloned into the plant binary expression vector pTRAkc (24) upstream and in-frame with codon-optimized hinge, C_H_2 and C_H_3 domains from human IgG1 (Fc1) (GenBank accession no. P01857; aa 99-330) and downstream of the signal peptide of the murine mAb24 heavy-chain (lph) (25). A flexible linker sequence, encoding (Gly-Gly-Gly-Gly-Ser)_2_, was included between the FH* and Fc to achieve separation of the functional domains (26). This construct was designated FH*/Fc1, and was described in a previous publication (22) by strain number (S2477).

A second construct reversed the positions of FH* and Fc1, encoding a protein with Fc1 at the N-terminal end and FH* at the C-terminal. This construct was designated Fc1/FH*, and was also designated by strain number (S2509).

A third construct replaced the IgG1 C_H_2 and C_H_3 domains of Fc1/FH* with a codon-optimized sequence encoding the corresponding domains of human IgG3 (GenBank accession no. CAA67886.1), with the R at position 435 (Eu numbering) replaced with H, conferring both longer *in vivo* half-life and Protein A binding (27). The construct retained the IgG1 hinge (rather than the IgG3 hinge), which had been shown to impart improved bactericidal activity of an IgG3 mAb against *N. meningitidis* (28). This construct was designated Fc3/FH*, or by strain number (S2534). Details of the three constructs used in this study is shown in **Table 1**.

**Table 1:** Description of plant-produced FH*/Fc molecules.

Strain	Designation	Modifications	Binary expression vector name
S2477	FH*/Fc1	TS-FH*-(GGGGS)_2_-IgG1 Fc	pTRAk-c-lph-(TS)FH*-(G_4_S)_2_-hFc1
S2509	Fc1/FH*	IgG1 Fc-(GGGGS)_2_-FH*	pTRAk-c-lph-hFc1-(G_4_S)_2_-(TS)FH*
S2534	Fc3/FH*	IgG3 Fc-(GGGGS)_2_-FH*	pTRAk-c-lph-hFc3(IgG1 hinge)(435H)-(G_4_S)_2_-(TS)FH*

Transient expression of recombinant proteins was accomplished by whole-plant vacuum infiltration (29) of *N. benthamiana* ΔXT/FT (30) using *A. tumefaciens* GV3101 (pMP90RK) (31) containing one of the binary expression vectors, co-infiltrated with *A. tumefaciens* GV3101 (pMP90RK) containing the binary vector pTRAkc-P19, encoding the post-transcriptional silencing suppressor P19 (32).

Leaves were collected 5-7 days after vacuum infiltration and frozen at -80°C until use. Purification of FH/Fc proteins was accomplished using a protocol previously used with another plant-produced Fc fusion (33), which incorporates affinity chromatography with Protein A-MabSelect SuRe or PrismA (GE HealthCare). Purified proteins were concentrated to ≥2 mg/ml using 30 kDa cut-off centrifugal concentrators, buffer exchanged into 5 mM glycine, 15 mM acetate, 80 mM NaCl, pH 5.0-6.0 and rendered sterile by filtration through 0.22 μm PES membrane filters. Protein concentrations were quantified using absorption at 280 nm and extinction coefficients predicted from the amino acid sequences.

Purified protein samples were analyzed using standard methods. Samples were subjected to SDS-polyacrylamide gel electrophoresis (under reducing and non-reducing conditions) on 4–20% Mini-PROTEAN^®^ TGX Stain-Free™ Protein Gels (Bio-Rad, Hercules, CA). Gel images were obtained using a Bio-Rad Gel Doc EZ imaging system.

Stability analysis of purified protein samples was carried out as follows. Samples were diluted in the indicated buffer (with added 0.025% sodium azide as preservative) to 0.5 mg/ml and stored in a humidified container at 37 °C or 40 °C. At the indicated time intervals, aliquots were removed and stored at -80 °C until analyzed by SDS-PAGE. SDS-PAGE StainFree analysis was carried out under non-reducing conditions and densitometry analysis was performed by BioRad Image Lab. First, we determined the % intact band (~88kD) over total visible bands in the given lane. Then, we determined the % intact band of a given time point relative to the corresponding Time 0 (T0) sample (stored at -80°C).

### Solid Fc3/FH* formulations prepared by lyophilization and spray drying

A stock solution of S2534 (Fc3/FH*) (8.39 mg/mL) in buffer consisting of 77 mM sodium chloride, 15 mM potassium acetate, and 5 mM glycine was thawed, and used to prepare three formulation solutions (A, B, and C) as described in **Table 2**. The solids composition for all three formulation solutions was ~ 1% (w/w). Trehalose and polysorbate 80 were obtained from Spectrum Chemical (Gardena, CA) in NF grade and used as received. The S2534 formulation solutions were filter sterilized (0.2 μm membrane filter) prior to lyophilization or spray drying. A 0.2 mL aliquot of each formulation in a microfuge tube was pre-frozen by plunging in liquid N_2_ and dried by lyophilization (Freezemobile 12SL, SP VirTis, Warminster, PA) to yield ~ 1.8 mg of a voluminous white solid containing 0.84 mg S2534 (Fc3/FH*). A 4 mL aliquot of each formulation was spray dried using a B-90 Nano Spray Dryer (Buchi, New Castle, DE) in the long cylinder configuration and the following conditions: medium nebulizer orifice (4 μm); gas flow rate = 130-135 L/min; inlet temperature = 100°C; outlet temperature = 35-39°C; spray rate = 45% (100 kHz); pressure = 45-50 mbar; sample pump rate = 30%.

**Table 2:** Composition of formulation solutions and resulting lyophilized and spray dried solids.

*Component*	*Formulation A*		*Formulation B*		*Formulation C*	
	*Solid*	*Solution*	*Solid*	*Solution*	*Solid*	*Solution*
NaCl	24.3%	0.22%	24.2%	0.22%	23.1%	0.22%
glycine	2.03%	0.02%	2.0%	0.02%	1.9%	0.02%
KCH_3_CO_2_	8.0%	0.07%	7.9%	0.07%	7.6%	0.07%
polysorbate 80	0.04%	0.0004%	0.50%	0.005%	5.1%	0.05%
trehalose	20.3%	0.19%	20.2%	0.19%	19.3%	0.19%
FH/Fc S2534	45.4%	0.42%	45.2%	0.42%	43.1%	0.42%
Total solids		0.92%		0.93%		0.97%

All composition given as % (w/w).

### Human complement

IgG- and IgM-depleted normal human serum (human complement) was purchased from Pel-Freez Biologicals (Rogers, Arkansas). Normal human serum (NHS) obtained from healthy volunteers (UMass Chan IRB Protocol H00007741) was used as a source of C4b-binding protein (C4BP) in flow cytometry experiments.

### Antibodies

Anti-human IgG–FITC and anti-mouse IgG-FITC were from Sigma-Aldrich and were used at a dilution of 1:100 in HBSS containing 0.15 mM CaCl_2_ and 1 mM MgCl_2_ (HBSS^++^) and 1% BSA (HBSS^++^/BSA) in flow cytometry assays. Goat anti-human FH, alkaline phosphatase conjugated anti-human IgG (Southern Biotechnology) and donkey anti-goat IgG were used in Western blots a dilution of 1:1000 in PBS with 5% non-fat dry milk. Anti-C4BP mAb 104 (34, 35) was used in flow cytometry assays at a concentration of 10 μg/mL.

### Flow cytometry

Binding of FH*/Fc chimeric proteins and C4BP to bacteria was measured by flow cytometry as described previously (21). Data were acquired on a BD LSR II flow cytometer, and data were analyzed using FlowJo software.

### Serum bactericidal assay

Serum bactericidal assays using bacteria grown in gonococcal liquid media supplemented with cytidinemonophospho-N-acetyl neuraminic acid (CMP-Neu5Ac) (2 µg/ml) were performed as described previously (21, 36). Approximately 2000 colony forming units (CFUs) of *N. gonorrhoeae* were incubated with 10% human complement [IgG and IgM depleted normal human serum (Pel-Freez)] in the presence or the absence of the FH*/Fc fusion protein (concentration indicated for each experiment). The final volume of the bactericidal reaction mixture was 150 µl. Aliquots of 25-µl reaction mixtures were plated onto chocolate agar in duplicate at the beginning of the assay (t_0_) and again after incubation at 37°C for 30 min (t_30_). Survival was calculated as the number of viable colonies at t_30_ relative to t_0_.

### Mouse strains

Human Factor H (FH) and C4b-binding protein (C4BP) (FH/C4BP) transgenic mice) in a BALB/c background have been described previously (37). FH/C4BP Tg mice express levels of FH and C4BP that are comparable to those found in human serum and show similar responses to a variety of stimuli as wild-type (wt) BALB/c mice (37).

### Mouse vaginal colonization model of gonorrhea

Use of animals in this study was performed in strict accordance with the recommendations in the *Guide for the Care and Use of Laboratory Animals* by the National Institutes of Health. The protocol (A-1717) was approved by the Institutional Animal Care and Use Committee at the University of Massachusetts Chan Medical School. Female mice 6–8 wk of age in the diestrus phase of the estrous cycle were started on treatment with 0.1 mg Premarin (Pfizer; conjugated estrogens) in 200 μl of water given s.c. on each of three days: −2, 0, and +2 (2 d before, the day of, and 2 d after inoculation) to prolong the estrus phase of the reproductive cycle and promote susceptibility to *N. gonorrhoeae* infection. In some experiments, slow-release estrogen pellets (β-estradiol 17-acetate 5 mg, released over 21 days; Innovative Research of America) were implanted subcutaneously on day -2. Antibiotics (vancomycin and streptomycin) ineffective against *N. gonorrhoeae* were also used to reduce competitive microflora (38). Mice were infected on day 0 with *N. gonorrhoeae* (strain and inoculum specified for each experiment). Mice were treated daily with FH*/Fc intravaginally (dose stated for each experiment) from day 0 until the conclusion of the experiment or were given a corresponding volume of PBS (vehicle controls).

### Statistical analysis

Concentration-dependent complement-mediated killing by FH/Fc across strains was compared using 2-way ANOVA. Absolute IC50 (FH*/Fc concentration predicted to yield 50% survival) was calculated with a best-fit curve using non-linear regression (GraphPad Prism 9). Experiments that compared clearance of *N. gonorrhoeae* in independent groups of mice estimated and tested three characteristics of the data (19, 21, 39): time to clearance, longitudinal trends in mean log_10_ CFU, and the cumulative CFU as area under the curve (AUC). Statistical analyses were performed using mice that initially yielded bacterial colonies on days 1 and/or 2. Median time to clearance was estimated using Kaplan–Meier survival curves; times to clearance were compared between groups using the Mantel–Cox log-rank test. Mean log_10_ CFU trends over time were compared between groups using 2-way ANOVA and Dunnett’s multiple comparison test. The mean AUC (log_10_ CFU versus time) was computed for each mouse to estimate the bacterial burden over time (cumulative infection). The means under the curves of two groups were compared using the nonparametric Mann–Whitney test because distributions were skewed or kurtotic. The Kruskal–Wallis equality-of-populations rank test was also applied to compare more than two groups in an experiment.

## Results

### Production of FH*/Fc chimeric molecules in Nicotiana benthamiana

**Figure 1:**
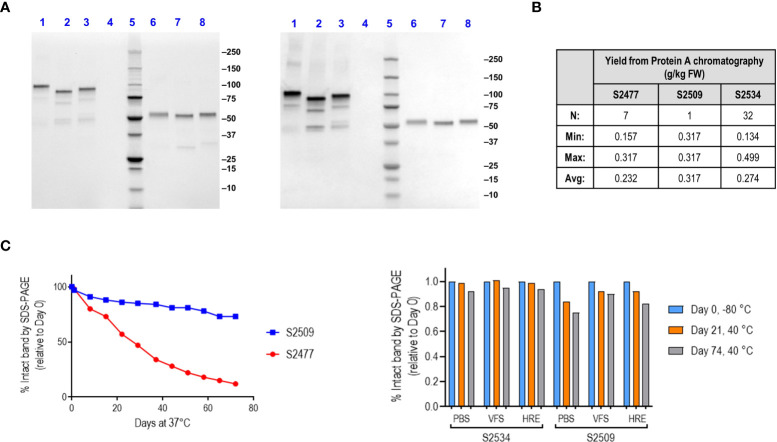
Production, characterization and stability of S2477 (FH*/Fc1), S2509 (Fc1/FH*) and S2534 (Fc3/FH*). **(A)**. SDS-PAGE visualized with StainFree (*Left image*) and western blots probed with anti-human IgG (*Right image*) of Protein A purified S2477 (FH*/Fc1) (lanes 1 and 6), S2509 (Fc1/FH*) (lanes 2 & 7), and S2534 (Fc3/FH*) (lanes 3 and 8) under non-reducing (lanes 1-3) and reducing conditions (lanes 6-8). **(B)**. Yields of the fusion proteins. The *Table* shows that yields of S2477 (FH*/Fc1) and S2534 (Fc3/FH*) purified by Protein A chromatography were similar over multiple processing runs. Only one processing run was carried out for S2509 (Fc1/FH*) but the observed yield was within the ranges observed for S2477 and S2534. **(C)** Stability of S2509 and S2477. *Left graph:* S2509 (Fc1/FH*) was more stable than S2477 (FH*/Fc1) over 76 days at 37°C. *Right graph:* Replacing human IgG1 Fc (S2509) with IgG3 Fc (S2534) improves the stability of Fc/FH* in three different buffers over 74 days at 40°C. VFS, simulated vaginal fluid that contains 77 mM NaCl, 7 mM lactic acid, 13 mM acetic acid, 25 mM glucose, 4 mM sodium acetate, 36 mM sodium lactate, pH 6. HRE (Histidine Arginine Glutamic acid buffer) contains 50 mM histidine, 36 mM arginine, 57 mM glutamic acid, 75 mM NaCl, pH 7.

Visualization of the proteins by StainFree SDS-PAGE gels and western blotting with goat anti-human IgG polyclonal antibody is shown in **Figure 1A**. Yield of S2477 and S2534 following Protein A affinity chromatography (over multiple purifications) ranged from 134-499 mg per kg plant fresh weight (**Figure 1B**). Only one run was performed for S2509, where yields were comparable with the two other proteins. Physical stability of FH*/Fc1 (S2477) and Fc1/FH* (S2509) was characterized by loss of the full-size intact band over up to 76 days at either 37°C or 40°C by scanning densitometry of StainFree SDS-PAGE gels (**Figure 1C**). Fc1/FH* (S2509) was more stable than FH*/Fc1 (S2477) (**Figure 1C**, left graph). Fc3/FH* (S2534) was even more stable than Fc1/FH* (S2509) (**Figure 1C**, right graph).

### FH* positioned C-terminal to Fc shows enhanced bactericidal activity

**Figure 2:**
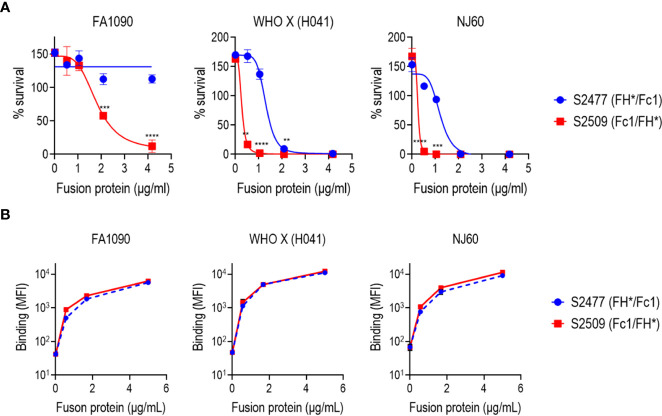
Bactericidal activity and binding of S2477 (FH*/Fc1) and S2509 (Fc1/FH*) produced in *N. benthamiana* against *N. gonorrhoeae in vitro*. **(A)** Serum bactericidal activity of S2477 (FH*/Fc1) and S2509 (Fc1/FH*). The proteins were incubated (concentrations indicated in the X-axis) with *N. gonorrhoeae* strains FA1090, WHO X (H041) and NJ60 and 20% human complement. Survival of bacteria following incubation for 30 min relative to CFU at 0 min is expressed as a percentage on the Y-axis. The mean (SEM) of 4 separate experiments is shown. The best-fit curve used to generate the IC50 is shown. Two-way ANOVA was used to measure differences in killing across the two molecules at each concentration. **P<0.01; ***P<0.001; ****P<0.0001. **(B)** Binding of S2477 (FH*/Fc1) and S2509 (Fc1/FH*) to *N. gonorrhoeae*. The fusion proteins (concentrations indicated on the X-axis) to *N. gonorrhoeae* FA1090, H041 and NJ60 was measured by flow cytometry. The median fluorescence intensity (MFI) is indicated on the Y-axis. The mean (range) of two separate experiments is shown.

Complement-mediated killing mediated by S2477 (FH*/Fc1) and S2509 (Fc1/FH*) against *N. gonorrhoeae* strains FA1090, WHO X (H041) and NJ60 were tested in serum bactericidal assays with 10% human complement (IgG/IgM depleted normal human serum) as the complement source. As shown in **Figure 2A**, S2509 showed improved activity against all three isolates. Of note, strain FA1090, which is highly resistant to killing by normal human serum (40) and also fully resistant to S2477 (>100% survival) (22), was killed 88% by S2509 at 4.17 µg/mL. The absolute IC50s (concentration of FH*/Fc calculated to yield 50% survival) for S2509 against FA1090, WHO X and NJ60 derived using nonlinear regression were 1.84, 0.25 and 0.18 µg/mL, respectively, while the IC50s for S2477 for WHO X and NJ60 were 1.33 and 1.04 µg/mL, respectively (IC50 for S2477 vs FA1090 by S2477could not be calculated because no killing was observed). Binding of S2477 and S2509 to all three strains by flow cytometry were similar (**Figure 2B**), suggesting that increased killing seen with S2509 was not attributable to increased binding.

### Efficacy of S2509 (Fc1/FH*) in the mouse vaginal colonization model

**Figure 3:**
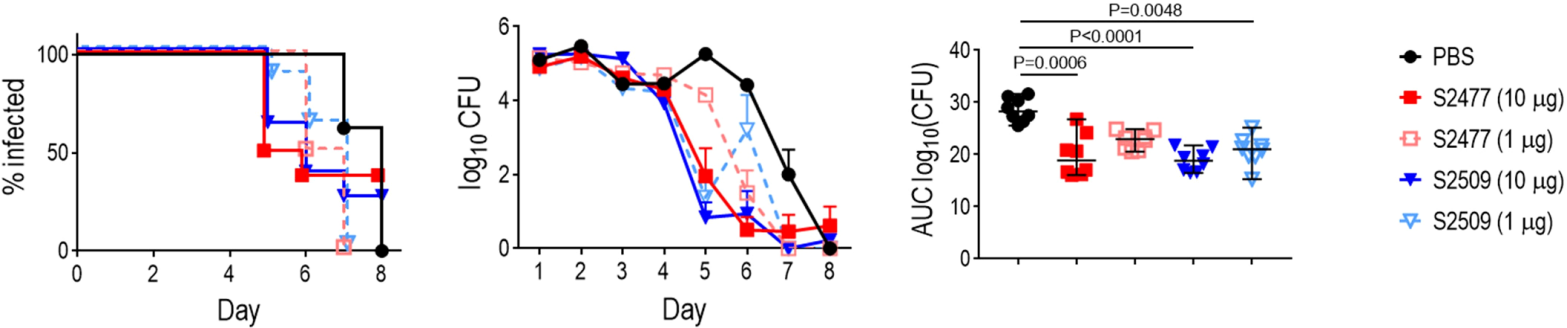
Efficacy of S2477 (FH*/Fc1) and S2509 (Fc1/FH*) against *N. gonorrhoeae* FA1090 in human FH/C4BP transgenic mice. Premarin^®^-treated 6-8 week-old human FH/C4BP transgenic mice (n=8/group) were infected with 2.8 x 10^7^ CFU *N. gonorrhoeae* FA1090. Mice were treated daily (starting 2 h before infection) intravaginally either with PBS (vehicle control) or with 1 µg or 10 µg of S2477 or S2509. *Left graph*: Kaplan Meier curves showing time to clearance, analyzed the Mantel-Cox (log-rank) test. *Middle graph*: log_10_ CFU versus time. X-axis, day; Y-axis, log_10_ CFU. Comparisons of the CFU over time between each treatment group and the respective saline control was made by two-way ANOVA and Dunnett’s multiple comparison test. All treatment groups showed statistically significantly lower CFUs (P<0.01) compared to the PBS group on day 5 and (with the exception of S2509, 1 µg/d) day 6. *Right graph*: Bacterial burdens consolidated over time (Area Under the Curve [log_10_ CFU] analysis). The AUC for each mouse was derived from the log_10_ CFU data shown in the middle graph. The five groups were compared by one-way ANOVA using the non-parametric Kruskal-Wallis equality of populations rank test. The χ^2^ with ties 22.77 (P = 0.0001). Pairwise AUC comparisons across groups was made with Dunn’s multiple comparison test.

The efficacy of S2509 (Fc1/FH*) against *N. gonorrhoeae* FA1090 *in vivo* was tested using the mouse vaginal colonization model (**Figure 3**); S2477 (FH*/Fc1) was used as a comparator. Mice were treated intravaginally, once daily, with either 1 or 10 µg of S2477 or S2509. While the times to clearance of the treatment groups did not differ significantly from the control group, both doses of S2509 and the 10 µg/d dose of S2477 showed significantly lower AUCs (a measure of overall bacterial burden over the course of the infection) compared to the control (PBS) group.

### IgG3 Fc improves the bactericidal activity of Fc/FH*

**Figure 4:**
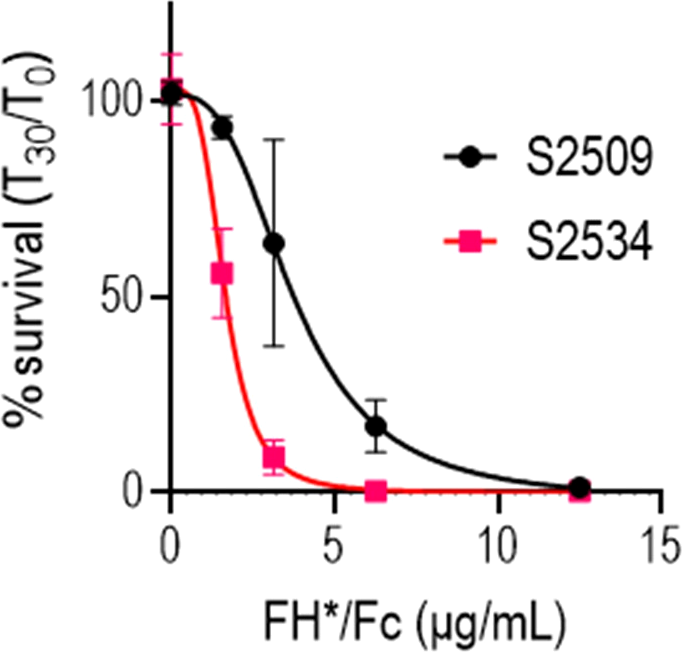
Replacing human IgG1 Fc with human IgG3 Fc improves bactericidal activity of FH*/Fc. *N. gonorrhoeae* FA1090 was incubated with increasing concentrations (indicated on the X-axis) of S2509 (Fc1/FH*) or S2534 (Fc3/FH*) and human complement (IgG/IgM depleted human serum) and survival at 30 min was measured (Y-axis). The mean (range) of 3 experiments is shown.

IgG3 is the most potent complement activator among human IgG subclasses (41). Using mice that lacked complement C6, we previously showed that killing mediated by membrane attack complex was essential for activity of FH*/Fc1 *in vivo* (22). Therefore, we asked whether activity of FH*/Fc could be further increased if IgG1 Fc in S2509 was replaced by human IgG3 Fc. As shown in **Figure 4**, S2534 (Fc3/FH*) showed a 56% decrease in the IC50 compared to S2509 against strain FA1090 (the IC50s for S2509 and S2534 were 3.71 and 1.64 µg/mL, respectively).

### Efficacy of S2509 and S2534 against a panel of 50 strains of *N. gonorrhoeae*

**Figure 5:**
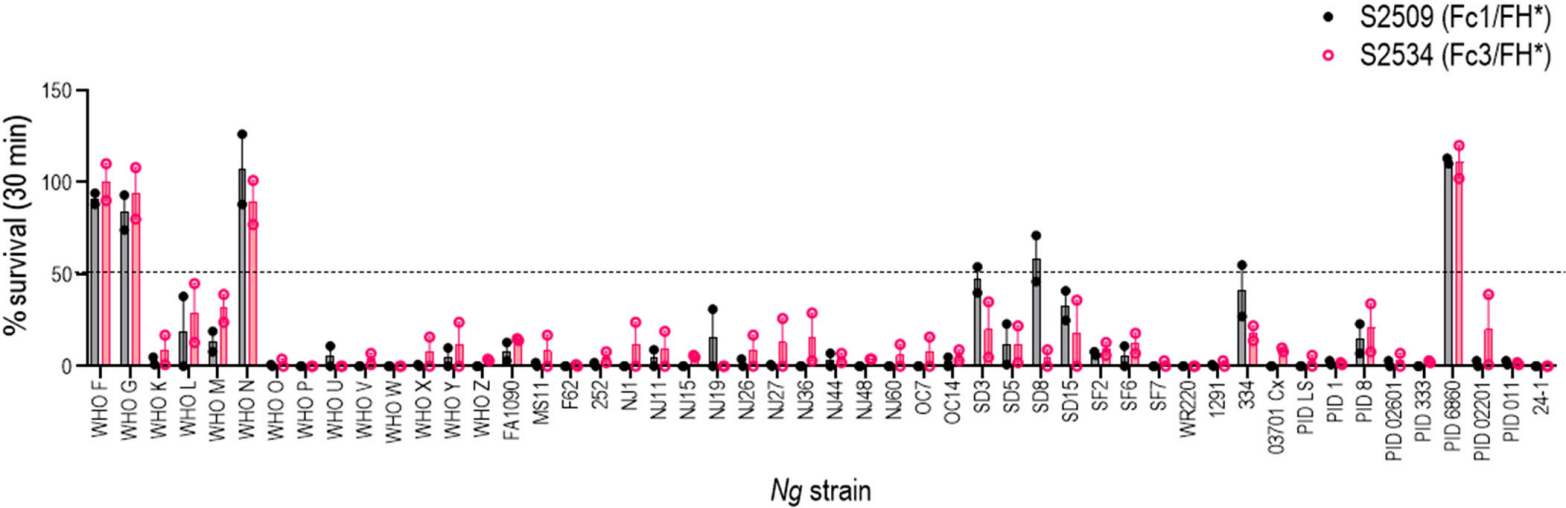
Complement-dependent bactericidal activity of S2509 (Fc1/FH*) and S2534 (Fc3/FH*) against a panel of 50 strains of *N. gonorrhoeae*. Fifty strains of *N. gonorrhoeae* (listed on the X-axis; see **Supplemental Table S1** for a description of strains) were incubated with the fusion proteins (33 µg/mL) and human complement (IgG and IgM depleted human serum; final concentration 10%). Bacterial survival at 30 min relative to 0 min is indicated as a percentage on the Y-axis. The mean (range) of two separate experiments is shown. Complement alone did not result in any killing of any strain (survival >100%) (data not shown).

**Figure 6:**
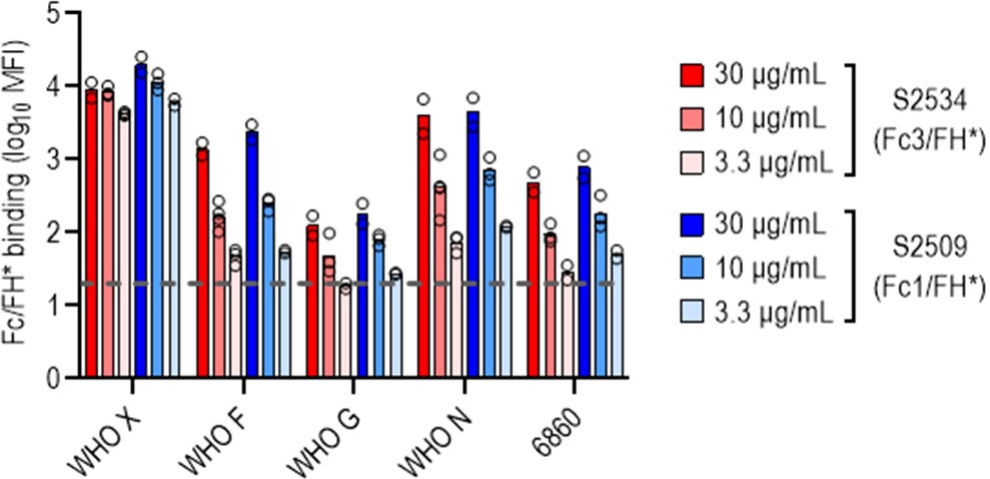
*N. gonorrhoeae* resistant to S2534 (Fc3/FH*) bind lower amounts of the fusion proteins. Binding of S2509 (Fc1/FH*) and S2534 (Fc3/FH*) to the four strains of *N. gonorrhoeae* that were resistant to killing by S2509 (>50% survival in **Figure 5**) was measured by flow cytometry. WHO X (H041) that was fully susceptible to S2509 and S2534 (100% killing) was included as a comparator. The Y-axis shows the log_10_ median fluorescence intensity (MFI) of fusion protein binding (mean and individual values of 2-4 separate experiments is shown). The dashed grey line indicates the average value of the conjugate controls (21; range from 11-37).

The bactericidal activities of S2509 (Fc1/FH*) and S2534 (Fc3/FH*) against 50 strains of *N. gonorrhoeae* were tested in a serum bactericidal assay. As shown in **Figure 5**, the activities of both molecules when tested at a dose of 33 µg/mL were similar; S2509 and S2534 showed activity (defined as >50% killing, or <50% survival) against 45/50 and 46/50 strains, respectively. We sought to determine whether the strains that survived >50% in the presence of S2534 (WHO F, WHO G, WHO N and 6860) bound less fusion protein than WHO X, a representative serum sensitive strain. We compared binding of S2509 and S2534 at concentrations of 30, 10 and 3.3 µg/mL to the five strains by flow cytometry. With the exception of WHO F and WHO N, which bound similar amounts of S2509 and S2534 as WHO X at 30 μg/mL, binding of both proteins to all resistant strains was significantly lower (P<0.05 by two-way ANOVA) than to WHO X at the corresponding protein concentrations (**Figure 6**).

### Reduced S2534 (Fc3/FH*) binding limits efficacy against PorB1A-exressing *N. gonorrhoeae*

**Figure 7:**
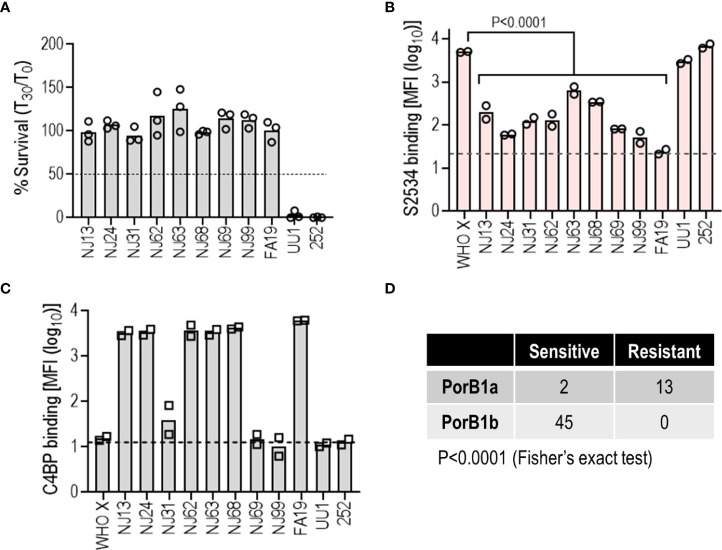
Low binding of S2534 (Fc3/FH*) limits its efficacy against PorB1A *N. gonorrhoeae*. **(A)** Complement-dependent bactericidal activity of S2534 (Fc3/FH*) against PorB1A gonococcal isolates. Ten additional PorB1A-expressing strains of *N. gonorrhoeae* (data with strain 252, used as a positive control for killing, has been shown in **Figure 6**) were incubated with S2534 (33 µg/mL) and human complement (IgG and IgM depleted human serum; final concentration 10%). Bacterial survival at 30 min relative to 0 min is indicated as a percentage on the Y-axis. The mean (range) of two separate experiments is shown. Complement alone did not result in any killing of any strain (survival >100%) (data not shown). **(B)** Binding of S2534 (Fc3/FH*; 3.3 µg/mL) to 11 PorB1A-expressing *N. gonorrhoeae* was measured by flow cytometry. WHO X (PorB1B) that was fully susceptible to S2534 (100% killing) was included as a comparator. The Y-axis shows the log_10_ median fluorescence intensity (MFI) of fusion protein binding (mean and individual values of 2 separate experiments). The dashed grey line indicates the average value of the conjugate controls (25.7). Comparisons across groups was made with one-way ANOVA and pairwise comparisons with WHO X were made by Dunnett’s multiple comparison test. **(C)** Binding of C4BP to *N. gonorrhoeae*. The 11 PorB1A *N. gonorrhoeae* strains shown in panel B and PorB1B strain WHO X were incubated with 10% normal human serum (NHS) as a source of C4BP. Bound C4BP was detected with mAb 104. Binding is expressed as log_10_ median fluorescence intensity (MFI) on the Y-axis (mean and individual values of two separate experiments). The dashed grey line indicates the average value of the conjugate controls (14.2). **(D)** The efficacy of S2534 correlates with the PorB molecule expressed. Bactericidal data from **Figures 6**, **7A** were compiled into the Table and analyzed with Fisher’s exact test.

*N. gonorrhoeae* strains contain a single *porB* gene in one of two allelic forms – *porB1B* or *porB1A* – that encodes Porin B (PorB), the major outer membrane protein (42). All four isolates in **Figure 6** that were resistant to killing by S2534 expressed the PorB1A allele. A fifth PorB1A isolate in that panel, strain 252 was susceptible to S2534. We therefore examined 10 additional gonococcal PorB1A-expressing isolates for susceptibility to complement-dependent killing by S2534 (**Figure 7A**). Strain 252 was included as a positive control for killing. Nine of the 10 strains were fully resistant (>95% survival); only strain UU1 in this expanded PorB1A panel was susceptible to S2534 at 33 µg/mL. The dose of S2534 was down-titrated in bactericidal assays for strains UU1 and 252 and revealed an IC50 of for 3.2 µg/mL for UU1. Strain 252 was killed 100% even at the lowest tested dose (0.5 µg/mL), thus the IC50 for this strain lay below 0.5 µg/mL.

We next tested binding of S2534 to these PorB1A strains. PorB1B strain WHO X was used as a comparator. S2534 was used at a concentration of 3.3 µg/mL, which revealed the greatest differences in binding across strains (**Figure 6**). As shown in **Figure 7B**, only the two sensitive PorB1A strains bound S2534 to the same extent as WHO X; all other resistant PorB1a isolates bound significantly lower amounts of S2534.

PorB1A strains tend to be intrinsically resistant to killing by nonimmune normal human serum (NHS), at least in part because these PorB1A strains bind the classical pathway inhibitor C4b-binding protein (C4BP) (40). While the two PorB1A strains that were sensitive to S2534 (strains UU1 and 252) did not bind C4BP as reported previously (40, 43), three isolates resistant to S2534 (NJ31, NJ69 and NJ99) also did not bind C4BP, suggesting that C4BP binding was not required for resistance to S2534 (**Figure 7C**).

Collectively, these data show that decreased binding of S2534 contributes to resistance of the PorB1A isolates. The susceptibility to S2534 of all strains shown in **Figuress 6**, **7** (45 PorB1b and 15 PorB1A) showed a strong correlation between PorB1A expression and resistance (**Figure 7D**).

### Efficacy of S2534 (Fc3/FH*) in the mouse vaginal colonization model of gonorrhea

**Figure 8:**
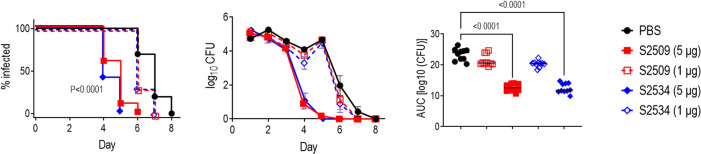
Efficacy of S2509 (Fc1/FH*) and S2534 (Fc3/FH*) against *N. gonorrhoeae* FA1090 in human FH/C4BP transgenic mice. Estrogen pellet-treated 6 week-old human FH/C4BP transgenic mice (n=10/group) were infected with 2.4 x 10^7^ CFU *N. gonorrhoeae* strain FA1090. Mice were treated daily (starting 2 h before infection) intravaginally either with PBS (vehicle control) or with 1 or 5 µg of each fusion protein. *Left graph*: Kaplan Meier curves showing time to clearance, analyzed the Mantel-Cox (log-rank) test. Both treatment groups that received 5 µg/d showed significantly faster clearance (P < 0.0001) compared to each of the other groups when compared in a pairwise manner. *Middle graph*: log_10_ CFU versus time. X-axis, day; Y-axis, log_10_ CFU. Comparisons of the CFU over time between each treatment group and the respective saline control was made by two-way ANOVA and Dunnett’s multiple comparison test. The two groups that received 5 µg of the fusion protein daily showed significantly lower CFUs compared to the PBS groups on days 4, 5 and 6 (P < 0.0001, P < 0.0001 and P < 0.05, respectively). *Right graph*: Bacterial burdens consolidated over time (Area Under the Curve [log_10_ CFU] analysis). The five groups were compared by one-way ANOVA using the non-parametric Kruskal-Wallis equality of populations rank test. The χ^2^ with ties was 39.11 (P < 0.0001). Pairwise AUC comparisons across groups was made with Dunn’s multiple comparison test.

**Figure 9:**
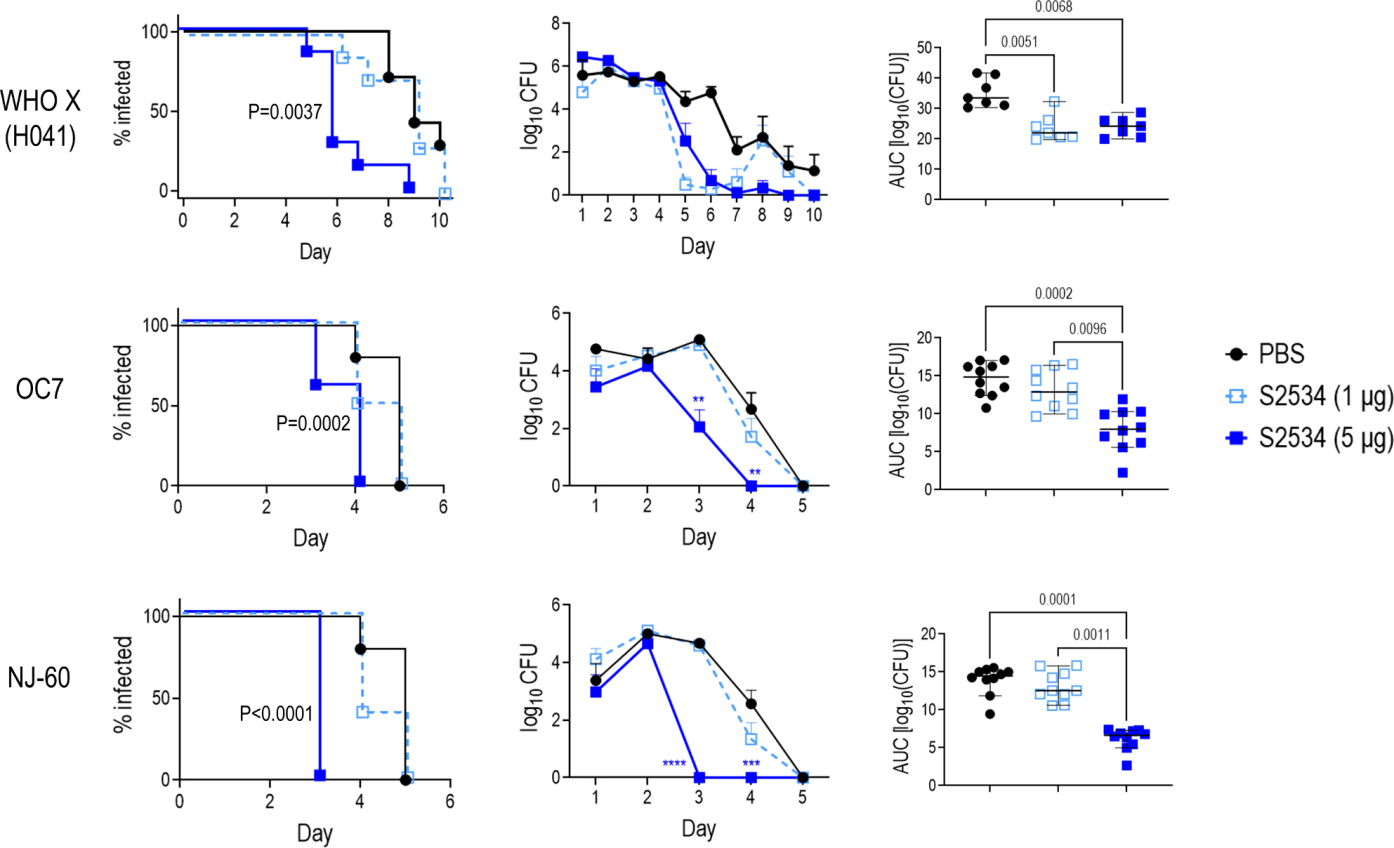
Efficacy of S2534 (Fc3/FH*) against three strains of *N. gonorrhoeae*. Premarin^®^-treated 6 week-old human FH/C4BP transgenic mice were infected with strains WHO X (H041) (*top row*; inoculum 2.5 x 10^7^ CFU; n = 7 mice/group), OC7 (*middle row*; inoculum 3.8 x 10^7^ CFU; n = 10 mice/group) or NJ60 (*bottom row*; inoculum 3.2 x 10^7^ CFU; n = 10 mice/group) and treated daily (starting 2 h before infection) intravaginally either with PBS (vehicle control) or with 1 or 5 µg of S2534. *Left graphs*: Kaplan Meier curves showing time to clearance, analyzed the Mantel-Cox (log-rank) test. P values indicate comparisons between the PBS and 5 µg groups. *Middle graphs*: log_10_ CFU versus time. X-axis, day; Y-axis, log_10_ CFU. Comparisons of the CFU over time between each treatment group and the respective saline control was made by two-way ANOVA. *Right graph*: Bacterial burdens consolidated over time (Area Under the Curve [log_10_ CFU] analysis). The three groups were compared by one-way ANOVA using the non-parametric Kruskal-Wallis equality of populations rank test. The χ^2^ with ties were 11.78 (P = 0.0007), 17.11 (P = 0.0002) and 19.61 (P < 0.0001) for WHO X (H041), OC7 and NJ60, respectively. Pairwise AUC comparisons across groups was made with Dunn’s multiple comparison test and the significant P values are indicated.

The efficacies of S2509 (Fc1/FH*) and S2534 (Fc3/FH*) against FA1090 were compared in the mouse vaginal colonization model. As shown in **Figure 8**, both molecules showed comparable efficacy by all three parameters (time to clearance, log_10_ CFU vs time and AUC analysis) when administered at a dose of 5 µg/d; both molecules were ineffective when given at a dose of 1 µg/d. To confirm efficacy *in vivo* against diverse clinical isolates, S2534 was tested in mice colonized with strains WHO X (H041; Japan), OC7 (California) or NJ60 (China) (**Figure 9**). S2534 was effective against all three isolates when given at a dose of 5 µg/d, while the 1 µg/d dose was effective only against strain WHO X.

### Fc3/FH* solid formulations retain activity *in vitro*

**Figure 10:**
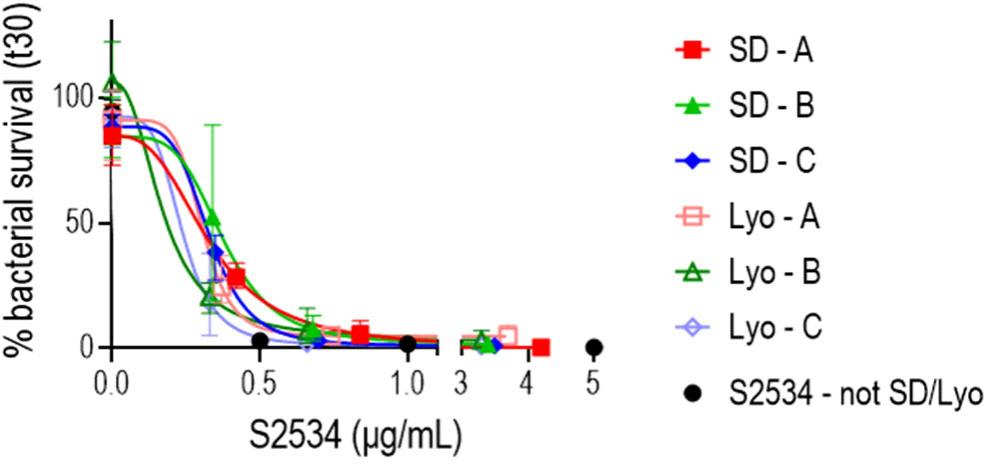
S2534 (Fc3/FH*) retains bactericidal activity after spray drying or lyophilizing. S2534 was spray-dried (SD) or lyophilized (Lyo) using three different formulations (A, B or C), as shown in **Table 2**. The bactericidal activity of each formulation reconstituted in water at concentrations of S2534 ranging from 0 to 5 µg/mL against strain FA1090 was determined. Each data point shows the mean (standard error) of 3 separate experiments. The calculated IC50 for SD – A, SD – B, SD – C, Lyo – A, Lyo – B and Lyo – C were 0.32, 0.38, 0.32, 0.29, 0.17 and 0.22 µg/mL, respectively. The IC50 for the native (not SD or lyophilized) S2534 could not be calculated because 97% killing was seen at the lowest concentration (0.5 µg/mL) tested. There were no statistical differences across the SD or Lyo groups by two-way ANOVA.

Incorporation of FH*/Fc molecules into a drug delivery device requires a stable formulation during both storage and use. Lyophilized or spray dried solid formulations are commonly used to protect proteins, including antibodies, in solid formulations that retain the biological activity of the protein as well as prevent aggregation and degradation (44–46). Three formulations of S2534 consisting of the Fc3/FH* protein in a matrix of sodium chloride and sodium acetate salts, the disaccharide trehalose, the amino acid glycine, and polysorbate 80 as a surfactant were prepared. Solid powders of each formulation were obtained by lyophilization and spray drying. As shown in in **Figure 10**, S2534 in reconstituted solutions prepared from spray dried and lyophilized powders of all three formulations retained bactericidal activity against FA1090.

## Discussion

*N. gonorrhoeae* has developed resistance to almost every antibiotic used for treatment and poses an urgent threat to human health worldwide. The “Global action plan to control the spread and impact of antimicrobial resistance in *N. gonorrhoeae*” highlights the need for novel approaches to prevent and treat gonorrhea (47). Complement is a critical arm of innate immune defenses against bacterial infections. Humans deficient in components of the alternative and terminal complement pathways are at a greatly increased risk of invasive Neisserial infections (13, 48, 49), including disseminated gonococcal infection (13, 49–54). The importance of complement-mediated lysis for the efficacy of an anti-gonococcal lipooligosaccharide antibody called mAb 2C7 and FH*/Fc1 at the mucosal surface was demonstrated in the gonococcal vaginal colonization model using mice deficient in terminal complement components C9 and C6 (*C9^-/-^* and *C6^-/-^* mice), respectively (22, 55).

Gonococci have evolved several strategies to escape complement, including binding human factor H (FH), a key inhibitor of the alternative pathway of complement (16). LOS sialylation also blocks binding of IgG against select surface antigens, thereby also inhibiting the classical pathway (56). Sialylation of gonococcal LOS occurs in humans (57) and during experimental infection of mice (58). Gonococci unable to sialylate their LOS are significantly impaired in their ability to colonize mice (58, 59).

In addition to inhibiting complement activation, LOS sialylation protects gonococci against killing by the cathelicidin LL-37 (60) and dampens the host inflammatory response by engaging immunoinhibitory Siglec receptors (61). Thus, targeting LOS sialylation with an immunotherapeutic is an attractive option. To achieve this, we designed an immunotherapeutic molecule combining the gonococcal-binding C-terminal domains 18, 19 and 20 of FH with human IgG1 Fc. Introducing a D-to-G mutation at position 1119 in FH domain 19 (FH*) abrogated lysis of human RBCs that was seen when unmodified FH domains 18-20 were fused to Fc, while retaining binding to and activity against gonococci *in vitro* and *in vivo* (21). Our lead Fc3/FH* molecule showed complement-dependent bactericidal activity against 92% of diverse gonococcal isolates tested.

Tobacco plants have been used for over three decades to produce antibodies and proteins (62). The tobacco plant expression system has advantages over mammalian cells because of the scalability of production, the potentially lower costs and the absence of animal viruses or prions (63). We previously showed that FH*/Fc1 produced in tobacco plants were effective against gonococci both *in vitro* and *in vivo* (22). Introducing a flexible linker between FH* and Fc1 improved bactericidal activity (22). In this study, we further improved the activity of FH*/Fc1 by placing the FH* fragment C-terminal to Fc and subsequently, by replacing human IgG1 Fc with IgG3 Fc. Interestingly, altering the orientation of FH* with respect to Fc (S2509 versus S2477) improved bactericidal activity *in vitro* without altering binding of the molecule. It is worth noting that S2509 was able to kill FA1090, a strain otherwise highly resistant to killing by normal human serum (40) that was also resistant to killing by previous iterations of FH*/Fc1, including S2477 (22). The mechanism of improved activity of S2509 over S2477, despite similar binding of both molecules to gonococci remains unclear. Given the importance of classical pathway activation in killing gonococci (64, 65), we speculate that S2509 engages C1q more effectively than S2477.

Consistent with their improved complement activity, the ‘reverse oriented’ molecules S2509 (Fc1/FH*) and S2534 (Fc3/FH*) showed activity against 100% of the 45 gonococcal isolates that expressed PorB1B. In comparison, an earlier study of FH*/Fc1 made in CHO cells showed bactericidal activity against only 10/15 (67%) of tested PorB1B isolates under similar assay conditions (21). The current and the previous studies both evaluated fusion protein activity versus the same 10 PorB1B-expressing isolates from Nanjing, China (strains with ‘NJ’ prefix); while all 10 strains were killed by S2509 and S2534 in this study, three strains (NJ11, NJ19 and NJ26) were resistant to the original FH*/Fc1 molecule (21). Collectively, this study provides compelling evidence for increased PorB1B strain coverage with the optimized Fc3/FH* molecule. However, Fc3/FH* could support killing of only 2 of 15 PorB1A strains tested, which could be attributed to decreased binding of Fc3/FH* to these strains. C4BP binding was not necessary for resistance of PorB1A isolates to S2534, evidenced by the three C4BP non-binding resistant isolates, NJ31, NJ69 and NJ99. Fc3/FH* can overcome complement inhibition by C4BP – an example is strain FA1090, a high C4BP binder that is otherwise highly resistant to normal human serum (40, 66).

The interaction of the C-terminus of FH with sialylated gonococci also requires concomitant expression of gonococcal PorB (67). While enhanced FH binding is seen when both, PorB1A and PorB1B strains are sialylated, the relative increase in FH binding seen following sialylation of PorB1A strains was less than that seen following sialylation of PorB1B isolates (67). Several PorB1A strains bind FH even when their LOS is not sialylated (68); FH binding in these strains may occur through domains 6 and 7 interactions with Neisserial surface protein A (NspA) (18).

The majority of strains isolated at local mucosal sites express PorB1B. The distribution of PorB1A vs PorB1B strains shows temporospatial differences. Of 110 isolates from individuals with uncomplicated gonococcal infection collected as part of the Gonococcal Isolate Surveillance Program from the Atlanta (Georgia) area in Georgia between 2017 and 2019, 91% (100/110) expressed PorB1B, while only 9% expressed PorB1A (69). Ninety-two of 103 isolates from remote regions in Russia isolated from 2004-2005 expressed PorB1B (70). Using serotyping methods, an analysis of 64 strains of *N. gonorrhoeae* in India collected between 2007 and 2008 showed that 26.5% reacted with anti-PorB1A antibodies, 48.4%with anti-PorB1B antibodies and 25% were recognized by both anti-PorB1A and PorB1B antibodies (71). A study of gonorrhea and chlamydia transmission in Boston in the 1990s showed that only 8 of 40 (20%) of strains from infected men expressed PorB1A (72). By contrast, PorB1A strains disproportionately represent isolates from persons with disseminated gonococcal infection (DGI). Two-thirds (20/30) of DGI isolates from Atlanta described by Cartee and colleagues were PorB1A (69). Of 49 DGI isolates in patients hospitalized at Boston City and University Hospitals over a 7-year period ending in 1982, 85% expressed PorB1A (73). PorB1A isolates represented 85 of 101 (84%) DGI isolates recovered from patients in the Seattle, Atlanta and Denver areas in the 1970s and early 1980s (74). While Fc3/FH* may provide near universal coverage against PorB1B isolates that are most commonly encountered, it has limited activity against PorB1A strains that have a greater capacity to cause DGI. Coverage of PorB1A strains will therefore require an alternative agent. A fusion protein comprising C4BP domains 1 and 2 that bind *N. gonorrhoeae* with IgM constant domains C_H_2, C_H_3 and C_H_4 showed activity against C4BP-binding gonococci, including ~90% of tested PorB1A strains (75). A fusion protein comprising FH domains 6 and 7 fused to IgG1 Fc also shows activity against *N. gonorrhoeae* PorB1B strains (19); testing efficacy of this molecule against PorB1A strains, which were not used in that study, is merited.

Improved complement-dependent bactericidal activity *in vitro* did not translate to improved efficacy *in vivo*. A possible reason is that the mouse model may lack the sensitivity to detect small differences in efficacy; further titration of dosing and larger numbers of mice per group may be needed to demonstrate superior efficacy *in vivo*. Using *C6^-/-^* mice, we showed previously that membrane attack complex was necessary for activity of FH*/Fc1 (22). Therefore, the serum bactericidal assay is likely a mechanistic correlate of protection of FH*/Fc, which leads us to posit that fusion proteins with enhanced complement activating properties will be advantageous *in vivo*.

A potential application for the fusion proteins is prevention of gonococcal infection in women by sustained delivery to the lower genital tract through an intravaginal ring (IVR). For more than 40 years, IVRs have been used for multiple indications, most notably contraception, and are a promising delivery system for drugs to treat or prevent sexually transmitted infections (76–79). IVRs are discrete, woman-controlled, and do not require a medical provider for placement. An IVR delivering the non-nucleoside reverse transcriptase inhibitor (NNRTI) dapivirine, when used consistently, was shown in two Phase 3 clinical trials to provide a significant 56% reduction in HIV infection (80, 81). Formulation of drugs into IVRs requires a stable formulation both in storage and during IVR use. Solid formulations of IgG prepared by lyophilization (82, 83) or spray-drying (84, 85) have been widely used to prepare stable solid protein formulations and should provide an ideal starting material to be used in IVR designs that utilize solid cores of active drug (86–88). Here, we show that Fc3/FH* retains its activity *in vitro* after lyophilization, an important step in its development as an IVR-delivered drug.

In summary, the optimized fusion proteins created in this study and expressed in tobacco plants show activity against a wide array of diverse gonococcal strains both *in vivo* and *in vitro* and may be amenable to formulation into IVRs to prevent gonorrhea in women. Targeting an important virulence factor such as sialic acid represents an innovative strategy to combat the global threat of multidrug-resistant gonorrhea.

## Data availability statement

The original contributions presented in the study are included in the article/**Supplementary Material**. Further inquiries can be directed to the corresponding author.

## Ethics statement

The animal study was reviewed and approved by Institutional Animal Care and Use Committee at the University of Massachusetts Chan Medical School.

## Author contributions

JS, AC, YT, LAL, JAM, KW, and SR contributed to the design of experiments, data analysis and writing of the manuscript. JS, AC, YT, BZ, NN, CS, RBD, SG, LAL, JM, and JAM performed the experiments. All authors contributed to the article and approved the submitted version.

## Funding

This work was supported by National Institutes of Health/National Institutes for Allergy and Infectious Disease grants R01AI132296, R44 AI147930 (to SR and KW) and R01 AI160247 (to SR and JM).

## Acknowledgments

We thank the reviewers for their insightful comments.

## Conflict of interest

Authors YT, JM, and KW were employed by Planet Biotechnology, Inc.

The remaining authors declare that the research was conducted in the absence of any commercial or financial relationships that could be construed as a potential conflict of interest.

## Publisher’s note

All claims expressed in this article are solely those of the authors and do not necessarily represent those of their affiliated organizations, or those of the publisher, the editors and the reviewers. Any product that may be evaluated in this article, or claim that may be made by its manufacturer, is not guaranteed or endorsed by the publisher.
