# Supplementary material for: An optimized Factor H-Fc fusion protein against multidrug-resistant Neisseria gonorrhoeae
Source: Front Immunol. 2022 Aug 30;13:975676. doi: 10.3389/fimmu.2022.975676 (PMC9468773; doi:10.3389/fimmu.2022.975676)
Supplement: Supplementary file 1 [file Table_1.docx]

**Supplemental Table S1.** Strains used in this study

| Strain | Relevant characteristics | Ref |
| --- | --- | --- |
| WHO F | NCTC no. 13477; PorB1a; MLST – ST10934; NG-MAST – ST3303 | 8 |
| WHO G | NCTC no. 13478; PorB1a; MLST – ST1903; NG-MAST – ST621 | 8 |
| WHO K | NCTC no. 13479; PorB1b; MLST – ST7363; NG-MAST -ST1424 | 8 |
| WHO L | NCTC no. 13480; PorB1b; MLST - ST1590; NG-MAST – ST1422 | 8 |
| WHO M | NCTC no. 13481; PorB1b; MLST – ST7367; NG-MAST – ST3304 | 8 |
| WHO N | NCTC no. 13482; PorB1a; MLST – ST1583; NG-MAST – ST556 | 8 |
| WHO O | NCTC no. 13483; PorB1b; MLST – ST1902; NG-MAST – ST495 | 8 |
| WHO P | NCTC no. 13484; PorB1b; MLST – ST8127; NG-MAST – ST3305 | 8 |
| WHO U | NCTC no. 13817; PorB1b; MLST – ST7367; NG-MAST – ST2382 | 8 |
| WHO V | NCTC no. 13818; PorB1b; MLST – ST10314; NG-MAST – ST8927 | 8 |
| WHO W | NCTC no. 13819; PorB1b; MLST – ST7363; NG-MAST – ST835 | 8 |
| WHO X | NCTC no. 13820; PorB1b; MLST – ST7363; NG-MAST – ST4220 | 8 |
| WHO Y | NCTC no. 13821; PorB1b; MLST – ST1901; NG-MAST – ST1407 | 8 |
| WHO Z | NCTC no. 13822; PorB1b; MLST – ST7363; NG-MAST – ST4015 | 8 |
| FA1090 | PorB1b; MLST - ST1899; NG-MAST – ST773 | 1 |
| FA19 | PorB1a MLST – ST1892; NG-MAST – ST13745 | 5 |
| MS11 | PorB1b MLST – ST6959; NG-MAST - ST4813 | 1 |
| F62 | PorB1b MLST – ST1900; NG-MAST – ST915 | 1 |
| 252 | PorB1a | 5 |
| NJ1 (UMNJ1) | PorB1b; MLST – ST11969; NG-MAST – ST3366 | 2 |
| NJ11 (UMNJ11) | PorB1b; MLST – ST 11173; NG-MAST – ST270 | 2 |
| NJ13 (UMNJ13) | PorB1a MLST – ST7367; NG-MAST – ST19720 | This study |
| NJ15 (UMNJ15) | PorB1b; MLST - ST1583; NG-MAST – ST19440 | 2 |
| NJ19 (UMNJ19) | PorB1b; MLST - ST11984; NG-MAST - ST19439 | 2 |
| NJ24 (UMNJ24) | PorB1a MLST - ST10633; NG-MAST - ST19721 | This study |
| NJ26 (UMNJ26) | PorB1b; MLST – ST1580; NG-MAST - ST11998 | 2 |
| NJ27 (UMNJ27) | PorB1b; MLST – ST7827; NG-MAST – ST2318 | 2 |
| NJ31 (UMNJ31) | PorB1a MLST – ST7356; NG-MAST - ST568 | This study |
| NJ36 (UMNJ36) | PorB1b; MLST – ST1600; NG-MAST – ST1766 | 2 |
| NJ44 (UMNJ44) | PorB1b; MLST – ST1901; NG-MAST - ST8736 | 2 |
| NJ48 (UMNJ48) | PorB1b; MLST – ST1583; NG-MAST – ST1053 | 2 |
| NJ60 (UMNJ60) | PorB1b; MLST – ST1600; NG-MAST – ST3289 | 2 |
| NJ62 (UMNJ62) | PorB1a MLST – ST1588; NG-MAST - ST12508 | This study |
| NJ63 (UMNJ63) | PorB1a MLST – ST1588; NG-MAST - ST12508 | This study |
| NJ68 (UMNJ68) | PorB1a MLST – ST7365; NG-MAST - ST18351 | This study |
| NJ69 (UMNJ69) | PorB1a MLST – ST7365; NG-MAST - ST18351 | This study |
| NJ99 (UMNJ99) | PorB1a: MLST – ST7822; NG-MAST - ST10335 | This study |
| OC7 | PorB1b; NG-MAST – ST8535 | 9 |
| OC14 | PorB1b; NG-MAST – ST3307 | 9 |
| SD3 | PorB1b; NG-MAST – ST2400 | 9 |
| SD5 | PorB1b; NG-MAST – ST1407 | 9 |
| SD8 | PorB1b; NG-MAST – ST2400 | 9 |
| SD15 | PorB1b; NG-MAST – ST1407 | 9 |
| SF2 | PorB1b; NG-MAST – ST1407 | 9 |
| SF6 | PorB1b; NG-MAST – ST8424 | 9 |
| SF7 | PorB1b; NG-MAST – ST8481 | 9 |
| WR220 | PorB1b | 6 and this study |
| 1291 | PorB1b; MLST – ST 8422; NG-MAST - 19075 | 7 |
| 334 | PorB1b | 4 |
| 03701 Cx | PorB1b | This study |
| PID LS | PorB1b | 4 and this study |
| PID1 | PorB1b; MLST 10154; NG-MAST - 19316 | 4 |
| PID8 | PorB1b | 4 |
| PID02601 | PorB1b | This study |
| PID333 | PorB1b | 4 and this study |
| PID6860 | PorB1a | 4 and this study |
| PID02201 | PorB1b | This study |
| PID11 | PorB1b | 4 and this study |
| 24-1 | PorB1b; MLST – ST8418, NG-MAST - 19315 | 4 |
| UU1 | PorB1a | 10 |

**References**

1. [A variable genetic island specific for Neisseria gonorrhoeae is involved in providing DNA for natural transformation and is found more often in disseminated infection isolates.](https://pubmed.ncbi.nlm.nih.gov/11454218/)

Dillard JP, Seifert HS. Mol Microbiol. 2001 Jul;41(1):263-77. doi: 10.1046/j.1365-2958.2001.02520.x. PMID: 11454218.

1. A Novel Factor H–Fc Chimeric Immunotherapeutic Molecule against Neisseria gonorrhoeae

Jutamas Shaughnessy, Sunita Gulati, Sarika Agarwal, Magnus Unemo, Makoto Ohnishi, Xia-Hong Su, Brian G. Monks, Alberto Visintin, Guillermo Madico, Lisa A. Lewis, Douglas T. Golenbock, George W. Reed, Peter A. Rice and Sanjay Ram. J Immunol January 15, 2016, 1500292; DOI: https://doi.org/10.4049/jimmunol.1500292

1. [Disseminated gonococcal infection: a prospective analysis of 49 patients and a review of pathophysiology and immune mechanisms.](https://pubmed.ncbi.nlm.nih.gov/6415361/) O'Brien JP, Goldenberg DL**,** Rice PA**.** Medicine (Baltimore). 1983 Nov;62(6):395-406. PMID: 6415361.
2. [Bactericidal antibody in genital infection due to Neisseria gonorrhoeae.](https://pubmed.ncbi.nlm.nih.gov/402426/)  Kasper DL, Rice PA, McCormick WM. J Infect Dis. 1977 Feb;135(2):243-51. doi: 10.1093/infdis/135.2.243. PMID: 402426
3. Binding of complement factor H to loop 5 of porin protein 1A: a molecular mechanism of serum resistance of nonsialylated Neisseria gonorrhoeae. Ram S, McQuillen DP, Gulati S, Elkins C, Pangburn MK, Rice PA. J Exp Med. 1998 Aug 17; 188(4):671-80.
4. Bactericidal antibody response of normal human serum to the lipooligosaccharide of Neisseria gonorrhoeae. [M A Apicella](https://pubmed.ncbi.nlm.nih.gov/?term=Apicella+MA&cauthor_id=3081657), [M A Westerink](https://pubmed.ncbi.nlm.nih.gov/?term=Westerink+MA&cauthor_id=3081657), [S A Morse](https://pubmed.ncbi.nlm.nih.gov/?term=Morse+SA&cauthor_id=3081657), [H Schneider](https://pubmed.ncbi.nlm.nih.gov/?term=Schneider+H&cauthor_id=3081657), [P A Rice](https://pubmed.ncbi.nlm.nih.gov/?term=Rice+PA&cauthor_id=3081657), [J M Griffiss](https://pubmed.ncbi.nlm.nih.gov/?term=Griffiss+JM&cauthor_id=3081657) PMID: **3081657** DOI: [10.1093/infdis/153.3.520](https://doi.org/10.1093/infdis/153.3.520)
5. Selection and immunochemical analysis of lipooligosaccharide mutants of Neisseria gonorrhoeae. [K C Dudas](https://www.ncbi.nlm.nih.gov/pubmed/?term=Dudas%20KC%5BAuthor%5D&cauthor=true&cauthor_uid=3123395) and [M A Apicella](https://www.ncbi.nlm.nih.gov/pubmed/?term=Apicella%20MA%5BAuthor%5D&cauthor=true&cauthor_uid=3123395). [Infect Immun.](https://www.ncbi.nlm.nih.gov/pmc/articles/PMC259310/) 1988 Feb; 56(2): 499–504.
6. The novel 2016 WHO Neisseria gonorrhoeae reference strains for global quality assurance of laboratory investigations: phenotypic, genetic and reference genome characterization. Magnus Unemo, Daniel Golparian, Leonor Sa´nchez-Buso’, Yonatan Grad, Susanne Jacobsson, Makoto Ohnishi, Monica M. Lahra, Athena Limnios, Aleksandra E. Sikora, Teodora Wi and Simon R. Harris. J Antimicrob Chemother 2016; 71: 3096–3108 doi:10.1093/jac/dkw288 Advance Access publication 17 July 20.
7. Gose, S., D. Nguyen, D. Lowenberg, M. Samuel, H. Bauer, and M. Pandori. 2013. Neisseria gonorrhoeae and extended-spectrum cephalosporins in California: surveillance and molecular detection of mosaic penA. BMC Infect. Dis. 13: 570.
8. Wetzler LM, Blake MS, Gotschlich EC. 1988. Characterization and specif icity of antibodies to protein I of Neisseria gonorrhoeae produced by injection with various protein I-adjuvant preparations. J Exp Med 168: 1883–1897. https://doi.org/10.1084/jem.168.5.1883.
